# Multi-Mycotoxin Contamination of Maize Silages in Flanders, Belgium: Monitoring Mycotoxin Levels from Seed to Feed

**DOI:** 10.3390/toxins13030202

**Published:** 2021-03-11

**Authors:** Jonas Vandicke, Katrien De Visschere, Maarten Ameye, Siska Croubels, Sarah De Saeger, Kris Audenaert, Geert Haesaert

**Affiliations:** 1 Department of Plants and Crops, Faculty of Bioscience Engineering, Ghent University, Valentin Vaerwyckweg 1, 9000 Ghent, Belgium; Jonas.Vandicke@UGent.be (J.V.); Kris.Audenaert@ugent.be (K.A.); 2 Biosciences and Food Sciences Department, Faculty Science and Technology, University College Ghent, Research Station HoGent-UGent, Diepestraat 1, 9820 Bottelare, Belgium; katrien.devisschere@hogent.be; 3 Department of Pharmacology, Toxicology and Biochemistry, Faculty of Veterinary Medicine, Ghent University, Salisburylaan 133, 9820 Merelbeke, Belgium; siska.croubels@ugent.be; 4 Department of Bio-analysis, Faculty of Pharmaceutical Sciences, Ghent University, Ottergemsesteenweg 460, 9000 Ghent, Belgium; Sarah.desaeger@ugent.be

**Keywords:** maize, mycotoxins, *Fusarium*, monitoring, forage, silage

## Abstract

Maize silage, which in Europe is the main feed for dairy cattle in winter, can be contaminated by mycotoxins. Mycotoxigenic *Fusarium* spp. originating from field infections may survive in badly sealed silages or re-infect at the cutting edge during feed-out. In this way, mycotoxins produced in the field may persist during the silage process. In addition, typical silage fungi such as *Penicillium* spp. and *Aspergillus* spp. survive in silage conditions and produce mycotoxins. In this research, 56 maize silages in Flanders were sampled over the course of three years (2016–2018). The concentration of 22 different mycotoxins was investigated using a multi-mycotoxin liquid chromatography-tandem mass spectrometry (LC-MS/MS) method, and the presence of DNA of three *Fusarium* spp. (*F. graminearum*, *F. culmorum* and *F. verticillioides*) was analyzed in a selection of these samples using quantitative polymerase chain reaction (qPCR). Every maize silage contained at least two different mycotoxins. Nivalenol (NIV) and deoxynivalenol (DON) were the most prevalent (both in 97.7% of maize silages), followed by ENN B (88.7%). Concentrations often exceeded the EU recommendations for DON and zearalenone (ZEN), especially in 2017 (21.3% and 27.7% of the maize silages, respectively). No correlations were found between fungal DNA and mycotoxin concentrations. Furthermore, by ensiling maize with a known mycotoxin load in a net bag, the mycotoxin contamination could be monitored from seed to feed. Analysis of these net bag samples revealed that the average concentration of all detected mycotoxins decreased after fermentation. We hypothesize that mycotoxins are eluted, degraded, or adsorbed during fermentation, but certain badly preserved silages are prone to additional mycotoxin production during the stable phase due to oxygen ingression, leading to extremely high toxin levels.

## 1. Introduction

Animal feed such as silage maize for dairy cattle may be contaminated with mycotoxins, secondary metabolites produced by a variety of fungi, which can cause severe acute and chronic toxic effects when ingested. Most mycotoxigenic fungi that grow on maize in the field cannot grow in postharvest silage conditions if the silage is compacted and sealed hermetically. For instance, two of the most common field infecting fungi, *Fusarium graminearum* and *Fusarium verticillioides*, grow optimally at a pH of 7 to 7.5 [1,2], while a well preserved silage reaches a stable pH between 3.7 and 4.2 [3,4,5]. This, in combination with low oxygen levels, leaves these *Fusarium* species unable to grow in a typical silage environment [5,6]. However, other fungal species such as *Penicillium* spp. and *Aspergillus* spp. are capable of surviving with lower oxygen and pH levels [7,8]. Furthermore, mycotoxins are stable molecules that may remain unchanged during the silage process [9,10,11,12], meaning that even in well-preserved silages without fungal activity, mycotoxins originating from the field can be detected, Lepom et al. (1988) [11] found that in naturally contaminated corn cob mix (CCM) silages the concentration of ZEN remained approximately constant over a 12-week test period, whereas *F. culmorum* could no longer be detected after 11 days, suggesting that ZEN was already produced before ensiling.

Some mycotoxins are more stable in silage conditions than others. Boudra et al. (2008) [12] found that the concentrations of DON, ZEN, FB1, and FB2 in small experimental silages decreased based on the molecules’ solubility characteristics, implying that water soluble mycotoxins might be eluted by fermentation effluent. Further reduction of mycotoxin concentrations in silages could be explained by microbial degradation or adsorption [13,14].

Next to mycotoxins produced in the field, additional production of mycotoxins in silage can occur in several ways. If a silage is not pressed and sealed correctly and oxygen remains present, *Fusarium* spores may germinate and colonize the maize silage and produce additional mycotoxins [15,16,17]. Some *Fusarium* species (e.g., *F. oxysporum*, *F. solani,* or *F. verticillioides*) are even able to survive at low oxygen levels (<0.5%) in vitro, so could potentially survive silage conditions as well [18,19,20,21,22]. Furthermore, during feed-out, new fungal spores may infect the silage via the cutting edge, or inactive fungal spores in the silage may be reactivated due to exposure to oxygen [8,23,24,25]. Besides (pre-)harvest management strategies to avoid mycotoxin contamination [26], at post-harvest measures can also be taken to minimize the risk of mycotoxin accumulation. Namely, a sufficient feed-out speed should be maintained to avoid excessive fungal growth and corresponding mycotoxin production [27,28]. Lastly, fungal species such as *Penicillium* spp. and *Aspergillus* spp. are well adapted to the silage conditions and may produce additional mycotoxins [7,8,9,29,30,31]. In the case of *Penicillium* spp., growth in silages happens in fungal hot-spots: layers or lumps of green-blue fungal biomass, mostly found near the top or sides of the silage. Farmers are routinely advised to remove these moldy hot-spots prior to feeding [32,33], in part to avoid mycotoxin intoxication. However, removing these hot-spots does not eliminate all mycotoxins from the silage. Concentrations of *Penicillium* mycotoxins, e.g., ROQ-C, MPA, etc., are significantly higher in these hot-spots [34,35,36], while other mycotoxins, e.g., DON, ZEN, etc., occur in equal or even in higher concentrations in other regions of the silage [13,37].

There have been many surveys of mycotoxins in different types of silages in the past, in various regions in the world [9,10,17,29,35,38,39,40,41,42,43,44,45,46,47,48,49,50,51,52,53]. For example, Gruber-Dorninger et al. (2019) [46] found in a global survey that 62% of the maize silages were contaminated with DON, 40% with ZEN, and 37% with FUMs. AFB1, OTA, and T2 were found in 6%, 6%, and 3% of the silages, respectively. Yet, none of the aforementioned surveys have researched the mycotoxin load from harvest until feed-out in silages in practice. Storm et al. (2014) [10] sampled 17 silage maize fields at harvest and 82 maize silages during feed-out in Denmark, but these samples did not originate from the same farms. González Pereyra et al. (2008) [54] sampled two silages in Argentina before and after fermentation, but only on four mycotoxins. Since most surveys detected large numbers of typical field mycotoxins in maize silage samples, it would be interesting to be able to follow the total mycotoxin content in maize from seed until feed.

The aim of this research was to investigate the mycotoxin load of maize silages in the north-western European region of Flanders (Belgium) over the course of three years. From 2016 until 2018, a total of 133 samples from 56 silages were gathered. Samples were analyzed for 22 different mycotoxins using a multi-mycotoxin liquid chromatography-tandem mass spectrometry (LC-MS/MS) method, and DNA of *F. graminearum*, *F. culmorum* and *F. verticillioides* was quantified using a quantitative polymerase chain reaction (qPCR) on a selection of these samples. Several silage quality parameters were determined. In addition, during harvest of maize fields with a known mycotoxin load [26], chopped maize mass was kept apart and placed in the silage in an open net bag. When the bag appeared during feed-out, subsamples were taken and the same analysis (LC-MS/MS, qPCR and silage quality) were performed. This allowed us to monitor the mycotoxin concentrations before and after ensiling.

## 2. Results

### 2.1. Mycotoxin Levels in Maize Silages in 2016–2018

Incidence, mean, median, and maximum concentrations of 133 maize silage cutting edge samples and the numbers of samples exceeding the European regulations can be found in Table 1. All raw data can be found in Supplementary Table S1. Net bag samples will be discussed later in Section 2.4.

NIV and DON were the most prevalent mycotoxins, both being present in 97.7% of the maize silage samples. The derivatives of DON, 3-ADON and 15-ADON, were found far less frequently (in 11.3% and 36.1%, respectively, of all samples), and their prevalence strongly depended on the year the silage was filled. In silages with maize from 2017, 3-ADON and 15-ADON were found in 29.8% and 66.0% of the samples, respectively, while in silages from 2016, none of the samples were contaminated with 3-ADON and only 12.2% with 15-ADON. The same trend could be found for ZEN, being detected in 74.5% of the samples in 2017, as opposed to only 4.1% in 2016. ENN B was the third most prevalent mycotoxin, with an occurrence of 88.7%. FUMs were not found in silages from 2016 but were detected in 23.4% of the samples in 2017 and contaminated almost two-thirds of the samples (64.9%) in 2018. ROQ-C was only found in 6.8% of the samples over the course of three years. NEO, FX, AFB1, AFB2, AFG1, AFG2, OTA, DAS, AOH, STERIG, and T2 were never detected.

Parallel to their incidence, the mean and median concentrations of DON, 3-ADON, 15-ADON, and ZEN were highest in 2017, with mean concentrations of 1334 µg/kg DM for DON and 449 µg/kg DM for ZEN. Maximum concentrations went as high as 8912 µg/kg DM for DON and 3124 µg/kg DM for ZEN, far higher than the EU regulations of 2000 µg/kg DM and 500 µg/kg DM for DON and ZEN respectively. [55]. Over the course of three years, 8.3% and 12.0% of the samples exceeded the EU regulations for DON and ZEN, resp. Especially in 2017, approximately one-quarter of all maize silage samples was contaminated with DON or ZEN in concentrations exceeding the EU regulations (21.3% and 27.7%, resp.). No samples exceeded the guidance values for FB1 + FB2, OTA, or T2, nor the maximum level for AFB1. The sample with the highest total mycotoxin load was contaminated with NIV, DON, 3-ADON, 15-ADON, ZEN, and ENN B, in a total concentration of 10,899 µg/kg DM (NIV not included).

**Table 1:** Mycotoxin contamination detected in maize silages in Flanders, Belgium, from 2016 until 2018.

**Mycotoxin**	**Positive Samples (%)**				**Mean Concentration ^a^ (µg/kg DM)**				**Median Concentration (µg/kg DM)**				**Max. Concentration (µg/kg DM)**				**Samples Exceeding EU Recommendation (%) ^b^**			
	**2016**	**2017**	**2018**	**2016–2018**	**2016**	**2017**	**2018**	**2016–2018**	**2016**	**2017**	**2018**	**2016–2018**	**2016**	**2017**	**2018**	**2016–2018**	**2016**	**2017**	**2018**	**2016–2018**
*n* samples	49	47	37	133	49	47	37	133	49	47	37	133	49	47	37	133	49	47	37	133
NIV ^c^	100	97.9	94.6	97.7	/	/	/	/	/	/	/	/	/	/	/	/				
DON	98.0	100	94.6	97.7	258	1334	370	670	201	621	222	287	742	8912	4466	8912	0	21.3	2.7	8.3
3-ADON	n.d.	29.8	2.7	11.3	n.d.	23	29	16	n.d.	0	0	0	n.d.	183	1080	1080				
15-ADON	12.2	66.0	29.7	36.1	6.3	137	49	64	0	80	0	0	108	520	285	520				
DON+ ^d^	98.0	100	97.3	98.5	265	1495	449	750	226	725	282	318	742	9583	5710	9583				
ZEN	4.1	74.5	32.4	36.8	11	449	129	199	0	225	0	0	367	3124	1428	3124	0	27.7	8.1	12.0
ENN B	95.9	85.1	83.8	88.7	115	78	62	88	63	66	52	57	658	396	353	658				
AME	14.3	14.9	2.7	11.3	23	16	5.8	16	0	0	0	0	264	154	214	264				
FB1	n.d.	23.4	64.9	26.3	n.d.	48	184	68	n.d.	0	71	0	n.d.	715	1871	1871	0	0	0	0
FB2	n.d.	4.3	27.0	9.0	n.d.	3.0	38	12	n.d.	0	0	0	n.d.	79	449	449	0	0	0	0
FB3	n.d.	n.d.	13.5	3.8	n.d.	n.d.	9.8	2.7	n.d.	n.d.	0	0	n.d.	n.d.	177	177				
FUM ^d^	n.d.	23.4	64.9	26.3	n.d.	51	232	82	n.d.	0	71	0	n.d.	795	2497	2497				
ROQ-C	10.2	6.4	2.7	6.8	49	18	0.4	24	0	0	0	0	1065	428	14	1065				
SUM	100	100	100	100	463	2107	877	1159	426	1303	609	625	1565	10,899	6329	10,899				

n.d.: Not detected. /: not quantified. ^a^ Arithmetic mean. ^b^ EU regulations: 2000 µg/kg for DON (complementary and complete feedstuffs for calves (<4 months)); 500 µg/kg for ZEN (complementary and complete feedstuffs for calves and dairy cattle); 20,000 µg/kg for FB1 + FB2 (calves (<4 months)); 250 µg/kg for T2 (compound feed) [55,56]. ^c^ Due to excessive interference by non-targeted compounds, reliable quantitative analysis of NIV could not be performed. However, its presence or absence could be established. ^d^ DON+ = the sum of the incidence/concentrations of DON, 3-ADON and 15-ADON; FUM = the sum of the incidence/concentrations of FB1, FB2 and FB3; SUM = The sum of the incidence/concentrations of all detected mycotoxins, except NIV.

**Figure 1:**
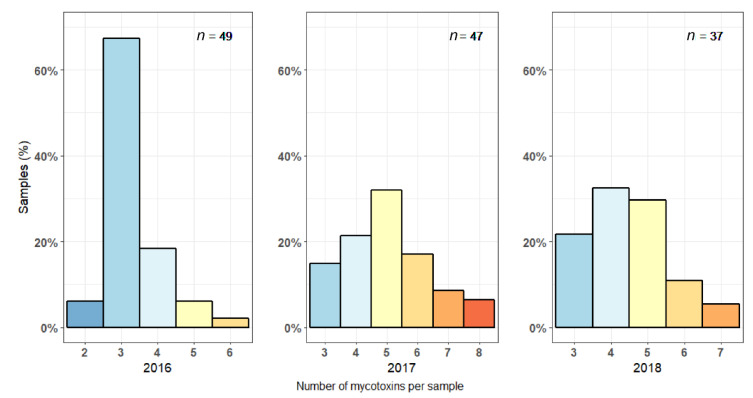
The relative number of maize silage samples contaminated with a certain number of different mycotoxins for 2016, 2017 and 2018.

Every silage sample in our survey was contaminated with at least two mycotoxins. The median load was four mycotoxins per sample, and more than one-third (38.3%) of all samples contained five or more different mycotoxins. Especially in 2017, silages were diversely contaminated, with samples containing up to eight different mycotoxins, and almost one-third (31.9%) of all samples containing six or more different mycotoxins (Figure 1). The median load per sample in 2017 was five, compared to three in 2016 and four in 2018.

### 2.2. Correlations between Different Mycotoxins

**Figure 2:**
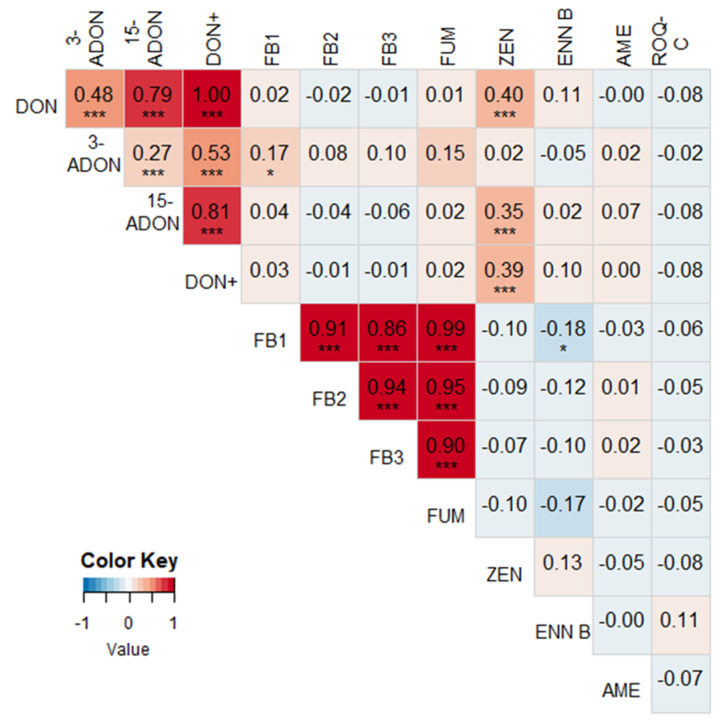
Heat map based on the pairwise Pearson correlation coefficients between the measured mycotoxin concentrations in maize silages in 2016–2018. A darker blue color indicates a stronger negative correlation, a darker red color indicates a stronger positive correlation. Significant correlations are indicated with asterisks (* = *P* < 0.05, *** = *P* < 0.01). DON+ = the sum of the concentrations of DON, 3-ADON, and 15-ADON. FUM = the sum of the concentrations of FB1, FB2, and FB3.

**Figure A1:**
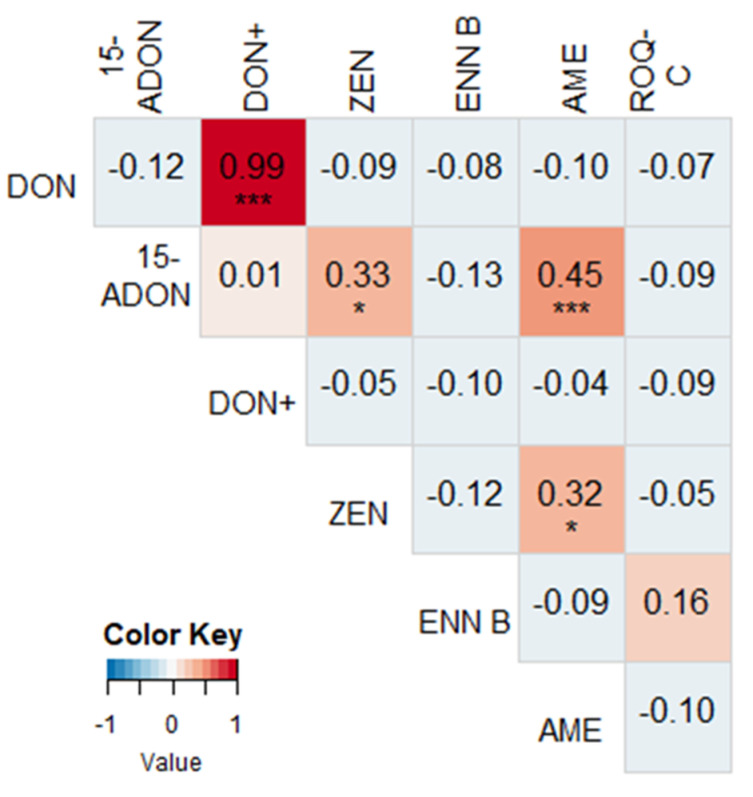
Heat map based on the pairwise Pearson correlation coefficients between the measured mycotoxin concentrations in maize silages in 2016. A darker blue colour indicates a stronger negative correlation, a darker red colour indicates a stronger positive correlation. Significant correlations are indicated with asterisks (* = *P* < 0.05, *** = *P* < 0.01). DON+ = the sum of the concentrations of DON, 3-ADON and 15-ADON.

**Figure A2:**
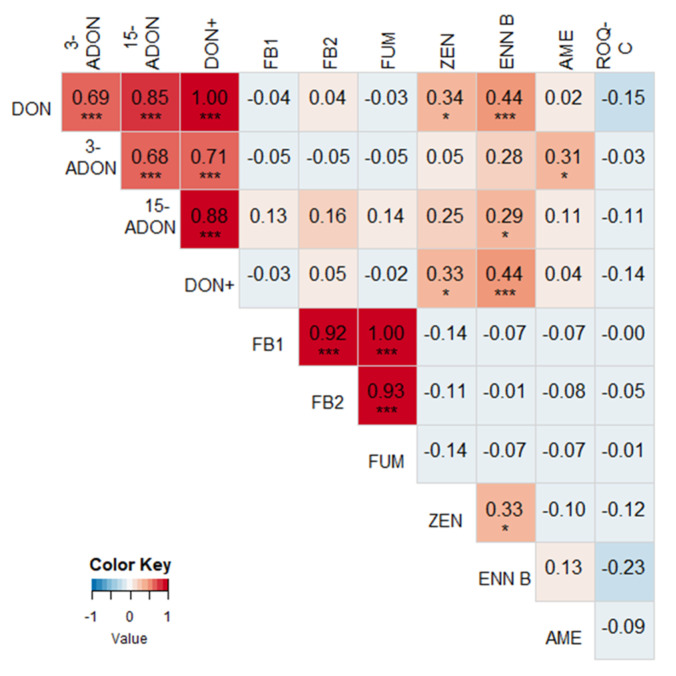
Heat map based on the pairwise Pearson correlation coefficients between the measured mycotoxin concentrations in maize silages in 2017. A darker blue colour indicates a stronger negative correlation, a darker red colour indicates a stronger positive correlation. Significant correlations are indicated with asterisks (* = *P* < 0.05, *** = *P* < 0.01). DON+ = the sum of the concentrations of DON, 3-ADON and 15-ADON. FUM = the sum of the concentrations of FB1, FB2 and FB3.

**Figure A3:**
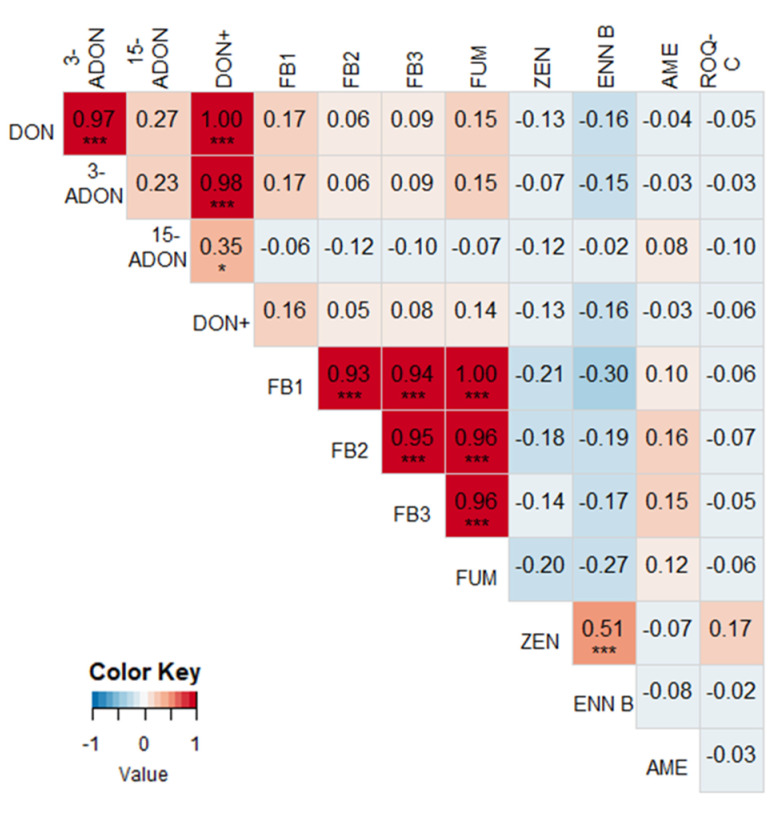
Heat map based on the pairwise Pearson correlation coefficients between the measured mycotoxin concentrations in maize silages in 2018. A darker blue colour indicates a stronger negative correlation, a darker red colour indicates a stronger positive correlation. Significant correlations are indicated with asterisks (* = *P* < 0.05, *** = *P* < 0.01). DON+ = the sum of the concentrations of DON, 3-ADON and 15-ADON. FUM = the sum of the concentrations of FB1, FB2 and FB3.

A heatmap with correlations between different mycotoxins for 2016–2018 is shown in Figure 2. ZEN was positively correlated with DON (*r* = 0.40, *P* < 0.001) and 15-ADON (*r* = 0.35, *P* < 0.001). Furthermore, a weak positive correlation between FB1 and 3-ADON (*r* = 0.17, *P* = 0.049) and a weak negative correlation between ENN B and FB1 (*r* = −0.18, *P* = 0.040) could be observed. As expected, DON and its derivatives (3-ADON and 15-ADON) and the FUMs (FB1, FB2, and FB3) were mutually positively correlated. No other significant correlations could be found. When splitting the data per year, new significant correlations came to light. For instance, in 2016 (Figure A1), AME shared a strong positive correlation with 15-ADON (*r* = 0.45, *P* = 0.001), as well as with ZEN (*r* = 0.32, *P* = 0.025). However, this is only based on seven positive samples for AME (out of a total of 49 samples in 2016). In 2017, ENN B was positively correlated with DON (*r* = 0.44, *P* = 0.002), 15-ADON (*r* = 0.29, *P* = 0.046), and ZEN (*r* = 0.33, *P* = 0.023) (Figure A2). In 2018, no significant correlations (besides DON and its derivatives and the FUMs) could be found, except between ZEN and ENN B (*r* = 0.51, *P* = 0.001) (Figure A3).

### 2.3. Correlations between Mycotoxin Concentrations and Fusarium spp. DNA

**Figure 3:**
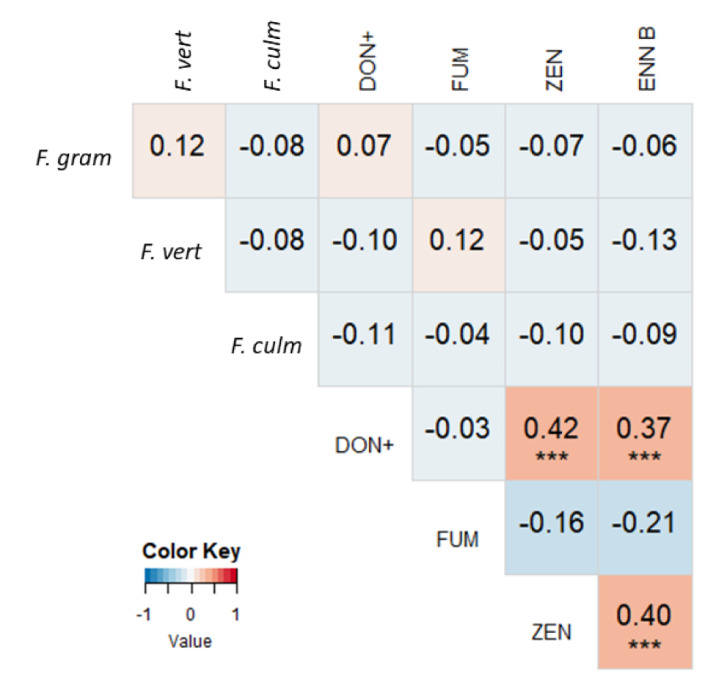
Heat map based on the pairwise Pearson correlation coefficients between measured mycotoxin concentrations and DNA of *F. graminearum*, *F. verticillioides,* and *F. culmorum* from 2017–2018. A darker blue color indicates a stronger negative correlation, a darker red color indicates a stronger positive correlation. Significant correlations are indicated with asterisks (* = *P* < 0.05, *** = *P* < 0.01). DON+ = the sum of the concentrations of DON, 3-ADON, and 15-ADON. FUM = the sum of the concentrations of FB1, FB2 and FB3.

Using qPCR analysis data, we could calculate correlations between mycotoxin concentrations and *Fusarium* spp. DNA in maize silages, and interspecies correlations between *Fusarium* species (Figure 3). These correlations are based on 48 samples from 2017 and 7 from 2018, so general conclusions for the three-year sampling period may not be drawn. After the removal of two outliers (more than 2.5 times the interquartile distance from the third quartile), not a single correlation could be found between DNA of three *Fusarium* spp., nor between *Fusarium* spp. and mycotoxin concentrations.

A summary of the frequency of detected *Fusarium* species and amount of fungal DNA can be found in Table A1.

### 2.4. Comparison between Mycotoxin Concentrations at Harvest vs. Net Bag Samples

No significant differences were found between cutting edge samples and net bag samples for any of the silage quality parameters (e.g., Flieg score, pH, ammonia content, etc.) (Table A2), meaning that the silage process in the net bags was similar to the fermentation in the silage around. For example, the average Flieg score of the net bag samples was 92.5, compared to 91.3 for the cutting edge samples (*P* = 0.374). Similarly, the mean concentration of each detected mycotoxin in the net bag samples was not significantly different compared to the cutting edge samples (data not shown), meaning that the net bags are representative for an average maize silage.

Table 2 shows the mean mycotoxin concentrations of the net bag samples (after ensiling) and the corresponding samples at harvest (before ensiling), and the mean difference between those two. A one-sample t-test with 0 as comparison value revealed that a significant decrease in the concentration after ensiling could be found for 3-ADON (*P* = 0.009), ZEN (*P* = 0.026). No significant increase or decrease in concentration was found for the other mycotoxins.

**Figure 4:**
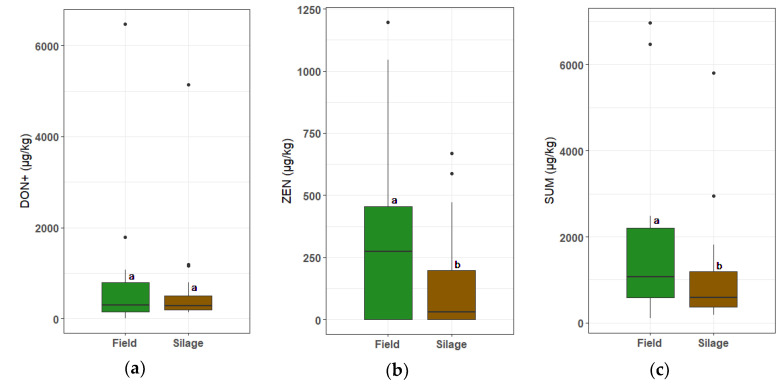
Concentrations of (**a**) DON, (**b**) ZEN, and (**c**) the total mycotoxin content in maize before (Field) and after ensiling (Silage). Concentration before ensiling is based on the known mycotoxin load of the respective maize field from Vandicke et al. (2019) [26]; Concentration after ensiling is based on the mycotoxin analysis of a net bag, filled with maize from that particular field and ensiled in a maize silage in practice. A significant difference before and after ensiling is indicated with significance letters (a, b). *n* = 22.

Despite rarely being significant, the results in Table 2 show that the mean concentration of every single detected mycotoxin in the net bags decreased after ensiling. In Figure 4, the concentrations pre- and post-fermentation are visualized for DON+, ZEN, and the total mycotoxin contamination, respectively. When looking at each individual case, we found that the concentration of DON, ZEN, and SUM after ensiling was reduced (or remained 0) in 55%, 86%, and 73% of the net bags, respectively.

## 3. Discussion

Maize silages in practice are very diverse. They often contain maize from several different fields. Furthermore, they can be filled in several ways: Some farmers are used to filling the trench silo layer per layer, while others prefer to push every new batch of harvested maize to the back of the trench silo. As a result, every trench silo has a unique, heterogeneous content, with a unique microbial community, and mycotoxin concentrations can change according to the width, height, depth, etc., of sampling. Furthermore, even in a hypothetical situation where a trench silo is completely homogeneous, silage quality on the top, bottom, and to the sides of the trench silo can be different than in the center [57,58]. In previous literature, several sampling techniques have been used depending on the research objectives, but no standard sampling technique has been adopted [13]. In this research, maize silages were sampled by taking eleven subsamples from fixed spots on the cutting edge of the silage. Fungal hot-spots were not targeted, nor avoided. This way, every part of the silage that is being fed to dairy cattle was sampled. One hundred and thirty-three samples from 56 maize silages were sampled this way over the course of three years. Furthermore, 22 net bags were ensiled, filled with maize from a specific field with a known mycotoxin load, enabling us to monitor changes in the mycotoxin concentrations before and after the ensiling process.

Every cutting edge sample contained at least two different mycotoxins. The mycotoxin load went as high as eight different mycotoxins in a single sample, indicating that multi-mycotoxin contamination in maize silages is very common. The fact that no maize silage is completely mycotoxin-free has been frequently described in the past, e.g., in Poland [41,47,52], Israël [53], and Serbia [45], among others. In an European survey on the presence of 61 mycotoxins in maize silages, Reisinger et al. (2019) found that an average maize silage was contaminated with 13 different mycotoxins, with 87% of the samples containing five or more mycotoxins.

NIV and DON were the most prevalent mycotoxins in our survey, followed by ENN B. DON is part of the multi-mycotoxin analysis in most surveys and has been found regularly in maize silages around the world [17,29,35,39,40,41,42,43,47,48,49,51,52,59]. In the global survey by Gruber-Dorninger et al. (2019) [46], DON was the most prevalent mycotoxin in maize silages worldwide, with a 62% incidence. NIV and ENN B on the other hand are rarely included in multi-mycotoxin analyses of maize silages, but are often among the most prevalent mycotoxins [10,29,41,50,53]. Grajewski et al. (2012) [52] found NIV in 88% of Polish maize silages between 2006 and 2009, being the second most prevalent mycotoxin (out of 13 analyzed) behind DON. In a survey of Polish maize silages in 2015, Panasiuk et al. (2019) [47] found that 86% of the samples contained ENN B, the second most prevalent mycotoxin after BEA, however mostly in low concentrations (<10 µg/kg DM). In our survey, the median concentration of ENN B was quite low as well (57 µg/kg DM). NIV was found in 54% of the samples. Lastly, Dagnac et al. (2016) [40] found ENN B to be the most prevalent mycotoxin (51%) in their two-year survey of 23 mycotoxins in Spanish maize silages. These results indicate that NIV and ENN B should be included in routine mycotoxin analysis of maize silages.

Some silages were contaminated with mycotoxins in concentrations that exceeded the EU regulations for DON and ZEN. Concentrations went as high as 8912 µg/kg DM for DON and 3124 µg/kg DM for ZEN. Over the course of three years, 8.3% of the silage samples exceeded the EU regulation for DON and 12.0% for ZEN. In previous literature, concentrations regularly exceeded the EU regulations for DON or ZEN in maize silages [29,39,40,41,42,45,46,48,49,52,59]. Pleadin et al. (2017) [59] found that 4.8% of maize silages in 2015 in Croatia exceeded the EU regulation for ZEN, and 9.5% for DON. Maximum concentrations of 7111 µg/kg (the UK [42]), 14,470 µg/kg (Poland [52]) and 34,861 µg/kg (global [46]) have been found for DON, and 3901 µg/kg (the UK [42]), 6239 µg/kg (global [46]) and 11,424 µg/kg (Croatia [59]) for ZEN. Several authors found maize silages that exceeded the EU regulation for AFB1, for instance in Serbia [45], Greece [60], and Brazil [39]. In our survey, no AFLAs were found. However, it is expected that climate change will cause tropical fungi such as *Aspergillus* spp. to move towards the poles [61] and invade temperate regions like Flanders [62,63,64].

A literature review by Ogunade et al. (2018) [65] revealed that in many cases the contribution of silage mycotoxins to the total amount of mycotoxins ingested by cows is greater than the maximum concentrations recommended by the EU or by the US FDA. As addressed by several authors [13,26,48,66], current legislation in the EU falls short, since multi-mycotoxin contamination and possible synergistic effects are not included. Furthermore, regulation is only set up for AFB1, DON, FB1 + FB2, OTA, ZEN, T2 + HT2, and ergot [55,56,67]. Frequently occurring or emerging mycotoxins such as NIV, ENN B, or DON derivatives are not included. Therefore, samples that do not exceed the current EU recommendations could still be toxic to dairy cattle.

A correlation study revealed that the concentrations of DON and ZEN in the maize silages in our survey were positively correlated. This relation has been found regularly in previous literature [41,47,48], but was not found in our previous study of freshly harvested maize in the same region and the same three years [26], except in 2017. ROQ-C was not correlated with any other mycotoxin, confirming that this mycotoxin is indeed typically formed in a silage environment, and that its production is therefore influenced by other factors. No correlations were found between different *Fusarium* spp., nor between *Fusarium* spp. and mycotoxin concentrations. This is an indication that the detected fungal DNA in silages and the corresponding mycotoxins did not originate from the silage but were already present at harvest. Cogan et al. (2017) [42] came to the same conclusion in their survey of grass and maize silages in England, where no relationship could be found between mould counts and mycotoxin concentrations. *Fusarium* spp. cannot survive typical silage conditions [5,11], and are hence rarely isolated from maize silages [9,30,51,68]. Tangni et al. (2017) [68] isolated more than 1000 different fungi from visually contaminated grass, maize, and sugar beet pulp silages in Belgium, and only 1% of these isolates were *Fusarium* spp. In this research, only *Fusarium* spp. were targeted.

The fact that no correlation could be found between mycotoxins and *Fusarium* spp. DNA in maize silages can also be explained by the role that certain mycotoxins play in the infection process. It is known that strains of *F. graminearum* that are unable to produce trichothecenes are less virulent than their mycotoxin-producing counter-parts on maize [69,70]. Trichothecenes aid in the infection process of *Fusarium* spp. by interfering with the plant’s defense system, hijacking the plants primary C and N metabolism and competing for space and nutrients with other organisms as an antibiotic or insecticide [71,72,73,74]. Since these traits are not needed for growth on harvested silage maize, a relation between *Fusarium* spp. DNA and trichothecenes in maize silages is less likely to be present, as was the case in our study. Why other mycotoxins such as FUMs are correlated with their main producer in the field but not in the trench silo is less clear. The exact biological role of many mycotoxins is still enigmatic.

In many ways, the mycotoxin content of maize silage cutting edge samples presented in this paper resembles the mycotoxin content of freshly harvested maize as described earlier in Vandicke et al. (2019) [26]. For instance, NIV remained the most prevalent mycotoxin; DON incidence and concentrations were highest in 2017; and the incidence of FUMs corresponded with their incidence in maize sampled at harvest, being most prevalent in 2018, occasionally found in 2017, and not detected in 2016. However, some differences between pre- and post-silage mycotoxin contamination can be found.

First, some mycotoxins that were found in maize at harvest between 2016 and 2018, i.e., AOH, DAS, FX, T2, and STERIG, were never detected in maize silages. Silages were consequently less diversely contaminated compared to maize at harvest: While the median mycotoxin load remained the same (four mycotoxins per sample), the maximum number of mycotoxins in one sample went from 10 at harvest [26] to 8 in silages (Figure 1). Grajewski et al. (2012) [52] found that T2 was present in 74% of Polish maize grain samples between 2006 and 2009, but only in 8% of maize silages. Similarly, Kosicki et al. (2016) [41] detected T2 in 67% of Polish maize samples between 2011 and 2014, compared to 48% in maize silages. In the global survey by Gruber-Dorninger et al. (2019) [46], the incidence of every analyzed mycotoxin (AFB1, FUMs, ZEN, DON, OTA and T2) was lower in maize silages compared to fresh maize. Incidence of FUMs for example decreased from 80% in maize to 37% in maize silages. Although exact figures were not available, these results indicate that the median mycotoxin load per sample was lower in maize silages compared to fresh maize, similar to our research. Certain mycotoxins that were found in low concentrations in freshly harvested maize were eluted or degraded to concentrations below the limit of detection, leading to less diversely contaminated silages.

Secondly, ROQ-C was found more frequently and in higher concentrations in maize silages than in freshly harvested maize (Table A3 and Table 1). This was expected, as ROQ-C is produced by *Penicillium* spp., a fungal genus generally considered to be a storage fungus. *Penicillium* was not included in our qPCR analysis so a relation between ROQ-C contamination and *Penicillium* spp. infection could not be confirmed; however, visual mold infestation could be seen on the cutting edge of all silages (except one) that were contaminated with ROQ-C. ROQ-C contamination was not severe, since only 6.8% of the maize silage samples contained ROQ-C. In a Danish research by Storm et al. (2014) [10], ROQ-C was only detected in 2 out of 82 maize silage samples. Shimshoni et al. (2013) [53] even found no ROQ-C in 15 Israeli maize silages. ROQ-C incidence and concentration much depends on the sampling zone. Since *Penicillium* spp. typically grow in hot-spots, ROQ-C concentrations in these hot-spots are far higher than in the rest of the silage [34,35,36,50]. Tangni et al. (2013) [34] found that the mean ROQ-C concentration in moldy maize silages in Belgium was four times higher than in non-moldy counterparts. Driehuis et al. (2008) [35] even found concentrations up to 45,000 µg/kg DM in maize silage hot-spots in the Netherlands. As explained above, in this research, we chose not to target these hot-spots and take a standardized sample of the entire cutting edge. This could explain the rather low incidence of ROQ-C in our survey.

Third, the maximum concentrations for some mycotoxins in maize silages were higher than those found in freshly harvested maize. The maximum concentration for DON went from 5322 to 8912 µg/kg DM, and from 2792 to 3124 µg/kg DM for ZEN (Table A3 and Table 1). Since the exact composition of every silage was not known, it is possible that certain silages contained highly contaminated maize from unknown fields from the start, and these concentrations remained the same throughout the silage process. Another possibility is that in certain silages, additional mycotoxins were produced. When a trench silo is not sealed off properly, Fusarium spores may germinate and colonize the maize silage and produce additional mycotoxins [15,16,17]. Furthermore, fungal spores may infect the cutting edge during feed-out [24]. This could explain why in the net bag samples, the mean mycotoxin concentrations decreased after ensiling (Table 2), and yet in some silages, the concentration increased (Figure 4). The use of net bags in silages is, to the best of our knowledge, unique, and provides the chance to monitor the mycotoxin contamination in maize from seed to feed.

It should be noted that mycotoxins can be bound or modified by plants (e.g., by conjugation to sugars) thereby rendering them less harmful. However, these masked mycotoxins can be transformed again to their toxic form during food/feed processing or digestion. As these masked mycotoxins are often not included in chemical analyses, reported mycotoxin content can represent an underestimation of actual levels [75]. In our study, we did not analyze these masked mycotoxins, even though they have already been reported in maize fields in Belgium and northern Germany, where the presence of the masked form was positively correlated with the parent compound [66,76]. Therefore, future analyses of maize silages should include these masked forms to better understand the kinetics of conjugated mycotoxins during the silage process and to better determine the risk for dairy cows.

We hypothesize that during the first phases of the silage process, the mycotoxin concentrations decreased by elution, degradation, or adsorption (for instance by lactic acid bacteria), and that during the stable phase certain aerated silages were re-infected and additional mycotoxins were produced. The latter part of this hypothesis has been observed in previous literature, where aerobic conditions in silages can cause an increase in the concentrations of AFLAs [23], FUMs [15,77], DON [77,78], and ZEN [77]. The first part of the proposed hypothesis, i.e., that the silage process reduces mycotoxin concentrations, is still up to debate [13]: Some researchers observed a decrease in the concentrations of the *Fusarium* mycotoxins DON, ZEN, and FB1 during ensiling [12,79], while others noticed no change or even an increase in concentration of the same mycotoxins [30,54,80]. These differing results are probably due to the fact that silage conditions differ greatly according to the DM, the epiphytic microorganisms, the length of storage, the initial air content, and the amount of air ingress, among others.

## 4. Conclusions

This research provides insight into the mycotoxin concentrations in maize silages in Flanders, and hence the possible mycotoxin load that is being fed to dairy cattle. NIV and DON were the most prevalent mycotoxins, both being present in 97.7% of the maize silages, followed by ENN B in 88.7%. The median mycotoxin load was four, with every silage containing at least two different mycotoxins. The mycotoxin contamination in silages was in many ways similar to that of freshly harvested maize. NIV and DON were present in nearly every silage, and the presence of FUMs was dependent on the sampling year, similar to the field. The presence of FUMs was favored by dry warm weather. However, certain typical silage mycotoxins such as ROQ-C were found more frequently and in higher concentrations than in the field. Furthermore, the maximum concentrations of certain field mycotoxins, e.g., DON and ZEN, were higher than those found in the field. Next, through the use of ensiled net bags filled with maize from a specific field, the evolution of mycotoxin concentrations could be investigated from seed to feed. These data revealed that the mean concentration of all detected mycotoxins decreased between harvest and feed-out. We hypothesize that mycotoxin concentrations are reduced during fermentation due to elution or degradation by microorganisms, but in certain silages, additional mycotoxins can be formed during the stable phase, leading to extremely high contaminations. The next step will be to identify which factors influence the course of the mycotoxin concentrations throughout all phases of the silage process.

## 5. Materials and Methods

### 5.1. Maize Silage Cutting Edge Sampling

**Figure 5:**
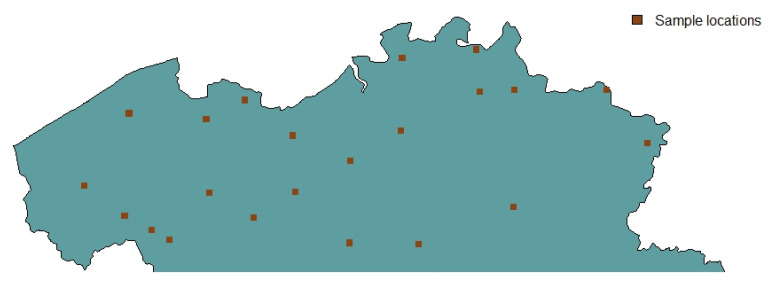
Location of the 22 maize silages in Flanders, Belgium.

A total of 106 dairy farmers across Flanders were contacted to participate in this study from 2016 until 2018, see also Vandicke et al. (2019) [26]. Based on an inquiry, we selected a total of 22 dairy farmers and sampled their maize silages from 2016, 2017, or 2018 (Figure 5). The selection was based on geography, type of silo (trench or ground silo), and other silage characteristics such as size, method of filling, feed-out speed, etc. Most farms had multiple maize trench silos per harvest year, and most trench silos (93%) contained maize from several different fields, but the sampled silage always contained maize from the field that was analyzed at harvest in the same year in our previous study [26]. The other fields in the trench silo originated from the same production area (same soil, weather conditions, etc.).

**Figure 6:**
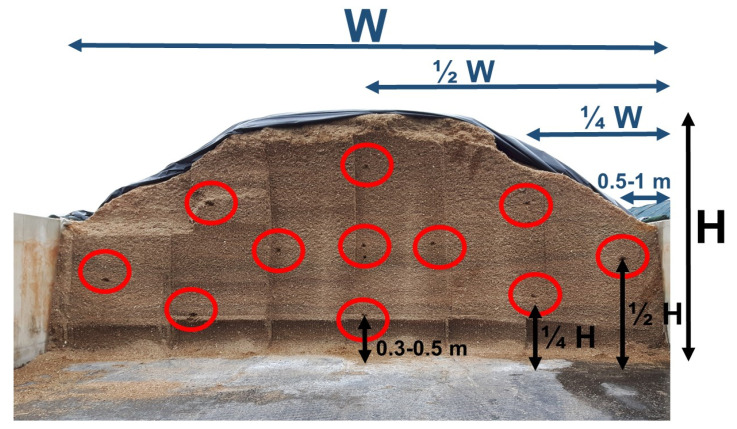
Sampling positions depicted on a maize silage. W = total width, H = total height (measured from the center of the silage).

Sampling was done by removing the first five centimeters of a specific spot on the cutting edge, and then taking ca. 50 g of maize silage. This process was repeated for a total of 11 different spots on the cutting edge, in a fixed pattern (Figure 6). This resulted in a maize silage sample of ca. 500 g. After sample collection, two subsamples were taken and stored in a freezer at −20 °C: A first subsample of ca. five grams for qPCR analysis (starting from 2017), and a second subsample of ca. 100 g to be sent to the Centre Provincial de l’Agriculture et de la Ruralité (CPAR) (La Hulpe, Belgium) for a silage quality analysis. The remaining sample was dried in an airstream of 65 °C for four days. The dried maize sample was then milled in a 0.5 mm sieve, and stored until further mycotoxin analysis. Ideally, this sampling process was performed three times throughout the feed-out process: At the start, in the middle and at the end of the silage. This was the case for most silages (51%), although some silages were only sampled one (14%) or two (35%) times. In three years, a total of 133 samples from 56 silages were gathered this way.

### 5.2. Maize Silage Density Sampling

Apart from the cutting edge sample, a sample was taken from the middle of the silage with a borer (Pioneer, 4.5 cm Ø, 45 cm long) to measure the density of the silage. The sample was dried at 65 °C for four days. Based on the fresh and dry weight of the sample and the volume of the borer, the density of the silage could be calculated. As the purpose of this sample was solely to calculate the density of the silage, no subsamples were taken and no further analysis was performed.

### 5.3. Net Bag Sampling

During harvest, all 22 farmers that were included in the maize silage sampling were asked to put aside some of the harvested maize from the selected field from our previous study [26], fill up an open net bag with this maize, and place it in the silage during filling. During feed-out, when the net bag appeared on the cutting edge, it was removed from the silage and stored at −20 °C. Further sample processing was similar to the cutting edge samples (i.e., taking subsamples for qPCR and silage quality analysis, drying, and milling). After a test case with only two farmers in 2016, a total of 22 net bag samples were collected over the course of three years.

### 5.4. Silage Quality Analysis

Several silage quality parameters were determined at CPAR. Nitrogen and ammonia content were determined according to Kjeldahl (1883) [81]. Based on these results, the fraction of ammonia nitrogen over total nitrogen was calculated. pH was determined by preparing an aqueous extract of the silage sample, after which pH was measured using a pH-electrode. The presence and quantity of the fermentation acids lactic acid, acetic acid, butyric acid, and propionic acid were determined by high-performance liquid chromatography (HPLC) according to Ohmomo et al. (1993) [82]. Flieg scores were calculated based on the lactic acid on total acidity ratio and the protein conservation [83].

### 5.5. Mycotoxin Analysis by LC-MS/MS

A list of the chemicals and procedures used for the quantification of the mycotoxins can be found in our previous study [26]. In short, a subsample of dried maize (2.5 g) was spiked with internal standards zearalanone (200 µg/kg) and deepoxy-deoxynivalenol (250 µg/kg), Subsamples were kept in the dark for 15 min and extracted with 20 mL of extraction solvent (acetonitrile/water/acetic acid (79/20/1, *v*/*v*/*v*)). After agitation on a vertical shaker for 1 h, samples were centrifuged for 15 min at 3300 g. Subsequently, the supernatant was passed through a preconditioned C18 solid phase extraction (SPE) column (Alltech, Lokeren, Belgium). The eluate was diluted to 25 mL with extraction solvent and defatted with 10 mL n-hexane. In order to recover all 22 mycotoxins, two different clean-up pathways were followed, see Vandicke et al. (2019) [26]. The samples were analyzed using a micromass Quattro Premier XE triple quadrupole mass spectrometer coupled with a Waters Acquity UPLC system (Waters, Milford, MA, USA). Data processing was done using the Masslynx^TM^ (4.1 version, Micromass, Manchester, UK) and Quanlynx^®^ software (4.1 version, Micromass, Manchester, UK). The analytical column used was a Symmetry C18, 5 µm, 2.1 × 150 mm, with a guard column of the same material (3.5 µm, 10 mm × 2.1 mm) (Waters, Zellik, Belgium) kept at room temperature. Liquid chromatography conditions and MS parameters were followed as described by Monbaliu et al. (2010) [84]. A list of the limits of detection and quantification can be found in Monbaliu et al. (2010) [85]. LC-MS/MS quality control was performed as described in [26].

### 5.6. qPCR Analysis

A quantitative PCR (qPCR) assay was used to quantify the total *F. graminearum*, *F. verticillioides* and *F. culmorum* DNA content in silage maize. Only a limited selection of 55 cutting edge samples (48 in 2017 and 7 in 2018) and 12 net bag samples (11 in 2017 and 1 in 2018) was analyzed using qPCR. The DNA extraction and qPCR analysis follows the procedure from [26]. In short, each subsample (5 g) was crushed with liquid nitrogen and 150 mg was transferred to a 1.5 mL Eppendorf tube for DNA extraction using a CTAB method modified for use with fungi [78]. The total amount of DNA was quantified with a Quantus fluorometer (Promega, Leiden, The Netherlands), and stored at –20 °C. Then qPCR analysis was performed (GoTaq^®^ qPCR Master Mix, Promega, Leiden, The Netherlands). The used primers were FgramB379 forward (CCATTCCCTGGGCGCT), FgramB411 reverse (CCTATTGACAGGTGGTTAGTGACTGG), FculC561 forward (CACCGTCATTGGTATGTTGTCACT), FculC614 reverse (CGGGAGCGTCTGATAGTCG), Fver356 forward (CGTTTCTGCCCTCTCCCA), and Fver412 reverse (TGCTTGACACGTGACGATGA) [86]. The qPCR analysis was performed using a CFX96 system (Bio-Rad, Temse, Belgium), including the following thermal settings: 95 °C for 3 min; 40 cycles of 95 °C for 10 s, and 60 °C for 30 s, followed by a dissociation curve analysis at 65 to 95 °C.

### 5.7. Statistical Analysis

The Pearson correlation coefficient was used to detect relations between different mycotoxins, between mycotoxins and fungal DNA, and between different *Fusarium* spp. at a significance level of *P* = 0.05. For calculation of the correlation coefficients, 3 outliers were discarded in the *F. verticillioides* DNA data. Differences in silage quality and mycotoxin contamination between net bag samples and cutting edge samples were investigated using a two-sample *t*-test. Significant differences between the mycotoxin concentrations before and after ensiling were investigated by calculating the difference between every coupled net bag sample and harvested maize sample, and performing a one-sample *t*-test with 0 as the comparison value. All statistical analyses were conducted using the R software package version 3.4.3 [87].

## Figures and Tables

**Table 2:** Mean mycotoxin concentrations in maize at harvest and after ensiling, and the mean difference before and after ensiling.

**Mycotoxin**	**Mean Concentration** **before Ensiling (µg/kg DM)**	**Mean Concentration** **after Ensiling (µg/kg DM)**	**Difference before−after** **Ensiling (%)**
DON	664 ± 241	532 ± 199	−19.9
3-ADON	49 ± 19	14 ± 9	−70.9 ***
15-ADON	90 ± 40	63 ± 22	−30.5
DON+ ^a^	803 ± 297	609 ± 225	−24.2
ZEN	326 ± 77	147 ± 45	−54.7 *
ENN B	67 ± 24	55 ± 16	−17.4
AME	16 ± 12	8.9 ± 6.2	−44.7
FB1	315 ± 204	155 ± 86	−50.9
FB2	94 ± 66	46 ± 29	−51.0
FB3	29 ± 21	12 ± 11	−59.4
FUM ^a^	437 ± 291	212 ± 125	−51.5

*n* = 22. Arithmetic mean values ± standard error of mean. A significant difference before and after ensiling is indicated with asterisks (* = *P* < 0.05, *** = *P* < 0.01). ^a^: DON+ = the sum of the concentrations of DON, 3-ADON and 15-ADON; FUM = the sum of the concentrations of FB1, FB2 and FB3.

## Data Availability

The data that support the findings of this study are available from the corresponding author upon reasonable request.

## Supplementary Materials

The following are available online at https://www.mdpi.com/2072-6651/13/3/202/s1, Table S1: Mycotoxin data maize silage flanders 2016–2018.

## Appendix A

**Table A1:** *Fusarium* spp. DNA detected in maize silages in Flanders, Belgium, from 2017 until 2018.

***Fusarium* spp. DNA**	**Positive Samples (%)**	**Mean Concentration ^a^ (pg/µL)**	**Median Concentration (pg/µL)**	**Max. Concentration (pg/µL)**
*F. graminearum*	81.4	0.105	0.032	1.530
*F. culmorum*	18.5	0.011	0	0.362
*F. verticillioides*	53.7	6.778	0.008	220.924

Total *n* = 55. 2017: *n* = 48; 2018: *n* = 7. ^a^ Arithmetic mean.

**Table A2:** Silage quality of cutting edge samples vs. net bag samples.

**Silage Quality Parameter**	**Mean in the Cutting Edge Samples (*n* = 115)**	**Mean in the Net Bag Samples (*n* = 115)**
pH	3.79 ± 0.01	3.83 ± 0.03
Ammonia content (g/kg DM)	0.888 ± 0.018	0.817 ± 0.076
Lactic acid content (g/kg DM)	48.73 ± 1.04	44.08 ± 2.82
Acetic acid content (g/kg DM)	15.05 ± 0.46	15.02 ± 1.58
Total Fliegscore	91.3 ± 0.5	92.5 ± 1.4

Arithmetic mean values ± standard error of mean. No significant differences were found between cutting edge samples and net bag samples for any of the silage quality parameters.

**Table A3:** Mycotoxin contamination detected in maize at harvest in Flanders, Belgium, from 2016 until 2018. Adapted from Vandicke et al. (2019) [26].

**Mycotoxin**	**Positive Samples (%)**				**Mean Concentration ^a^ (µg/kg)**				**Median Concentration (µg/kg)**				**Max. Concentration (µg/kg)**				**Samples Exceeding EU Recommendation (%) ^b^**			
	**2016**	**2017**	**2018**	**2016–2018**	**2016**	**2017**	**2018**	**2016–2018**	**2016**	**2017**	**2018**	**2016–2018**	**2016**	**2017**	**2018**	**2016–2018**	**2016**	**2017**	**2018**	**2016–2018**
*n* samples	91	81	85	257	91	81	85	257	91	81	85	257	91	81	95	257	91	81	95	257
NIV	98.9	100	98.8	99.2	651	719	882	749	528	461	782	587	2368	6776	2410	6776				
DON	92.3	100	64.7	85.6	449	558	187	396	263	337	121	215	2777	5322	2111	5322	2.2	3.7	1.0	2.3
3-ADON	78.0	29.6	15.3	42.0	54	36	23	38	43	0	0	0	297	380	1047	1047				
15-ADON	64.8	51.3	12.9	43.4	95	81	15	65	71	18	0	0	819	769	249	819				
DON+ ^c^	95.6	100	64.7	86.8	598	675	225	499	377	407	130	262	3050	6472	2111	6472				
ZEN	64.8	40.7	42.4	49.8	101	159	176	160	71	0	0	0	1086	1412	2792	2792	1.1	8.6	12.6	7.8
ENN B	42.9	18.5	45.9	36.2	133	78	180	150	56	28	71	46	1375	1042	1985	1985				
AOH	n.d.	3.7	9.4	4.3	n.d.	1.4	6.5	2.6	n.d.	0	0	0	n.d.	49	209	209				
AME	2.2	3.7	10.6	5.4	0.8	12	20	11	0	0	0	0	49	371	453	453				
FB1 ^d^	2.5	19.8	61.2	28.6	1.5	61	247	107	0	0	54	0	70	1363	4415	4415	0	0	0	0
FB2 ^d^	n.d.	4.9	24.7	10.2	n.d.	9.0	62	24	n.d.	0	0	0	n.d.	413	1427	1427	0	0	0	0
FB3 ^d^	n.d.	7.4	18.8	9.0	n.d.	3.4	18	7.4	n.d.	0	0	0	n.d.	91	451	451				
FUM ^c^	2.5	19.8	61.2	28.6	1.3	74	327	132	0	0	59	0	70	1783	6294	6294				
DAS	11.0	8.6	5.9	8.6	0.3	0.3	0.4	0.4	0	0	0	0	6.1	10	15	15				
FX	n.d.	7.4	2.4	3.1	n.d.	14	2.7	5.4	n.d.	0	0	0	n.d.	224	162	224				
T2	1.1	n.d.	8.2	3.1	0.2	n.d.	6.2	2.1	0	n.d.	0	0	17	n.d.	122	122	0	0	0	0
STERIG	1.1	n.d.	1.2	0.8	0.2	n.d.	2.6	0.9	0	n.d.	0	0	15	n.d.	73	205				
ROQ-C ^d^	n.d.	2.5	2.9	1.7	n.d.	0.6	0.6	0.4	n.d.	0	0	0	n.d.	30	25	30				
TOTAL ^c^	98.9	100	100	100	1485	1730	1877	1692	1310	1088	1596	1310	4153	13,748	8309	13,748				

n.d.: Not detected. ^a^: Arithmetic mean. ^b^: EU regulations: 2000 µg/kg for DON (complementary and complete feedstuffs for calves (<4 months)); 500 µg/kg for ZEN complementary and complete feedstuffs for calves and dairy cattle; 20,000 µg/kg for FB1 + FB2 (calves (<4 months)); 250 µg/kg for T2 (compound feed) [55,56]. ^c^: DON+ = the sum of the incidence/concentrations of DON, 3-ADON and 15-ADON; FUM = the sum of the incidence/concentrations of FB1, FB2 and FB3; TOTAL = The sum of the incidence/concentrations of all detected mycotoxins. ^d^: In 2016, only 79 samples were analysed for FB1, FB2 and FB3. In 2018, only 68 samples were analysed for ROQ-C.
